# Changing Template/Al_2_O_3_ Ratio in Reaction Gel—An Effective Way to Regulate Nature of Intermediate Phases and Properties of SAPO-11 Molecular Sieves during Crystallization

**DOI:** 10.3390/ma17061359

**Published:** 2024-03-16

**Authors:** Marat R. Agliullin, Dmitry V. Serebrennikov, Boris I. Kutepov

**Affiliations:** Ufa Federal Research Centre of the Russian Academy of Sciences (UFRC RAS), Institute of Petrochemistry and Catalysis, 450075 Ufa, Russia

**Keywords:** intermediate phases, SAPO-11 silicoaluminophosphate, catalysts, hydroisomerization n-hexadecane

## Abstract

A study has been conducted to investigate the formation of intermediate phases during the crystallization of SAPO-11 molecular sieves from reaction mixtures with a varying template (di-n-propylamine) DPA/Al_2_O_3_ ratio. It was found that changing the DPA/Al_2_O_3_ ratio from 1.0 to 1.8 in the initial reaction gels leads to the formation of different intermediate phases during crystallization into a SAPO-11 molecular sieve. It is shown that at the ratio template/Al_2_O_3_ = 1.0, an intermediate amorphous silicoaluminophosphate is formed; at 1.4, a mixture consisting of amorphous and layered phases forms; and at 1.8, a layered phase is present. A simple and innovative approach for controlling the morphology, size, and characteristics of primary crystals and the secondary porous structure in hierarchical SAPO-11 is proposed. The method is based on regulating the DPA/Al_2_O_3_ ratio in the reaction gel. The synthesized SAPO-11 molecular sieves with a hierarchical porous structure exhibited high selectivity in the hydroisomerization of n-hexadecane.

## 1. Introduction

Zeolites (molecular sieves) are one of the crucial components in the development of advanced adsorbents and catalysts for the chemical industry [1,2]. As a rule, most of the research in the field of zeolites is devoted to aluminosilicates; at the same time, in recent decades, new promising catalysts and adsorbents, which include silicoaluminophosphate molecular sieves SAPO-n, have appeared [3,4]. Thus, industrial catalysts for the production of lower olefins from methanol (MTO) and the isodewaxing of diesel fuels and oils based on SAPO-34 and SAPO-11 silicoaluminophosphate have proven to be significant [5,6].

SAPO-n is a wide class of microporous materials, the pore size of which can vary from 3.8 Å to 12.7 Å, and the organization of the channel system in space can be 1-dimensional, 2-dimensional or 3-dimensional [7]. The Brønsted acid sites within SAPO-n are formed during the incorporation of silicon into the aluminophosphate framework, and a negative charge is formed on the bridging oxygen atom, which is compensated by a proton. Currently, two mechanisms of silicon SM (substitution mechanism) introduction into the lattice have been confirmed [3]. In accordance with the SM2 mechanism, silicon atoms undergo isomorphic substitution for phosphorus atoms within the aluminophosphate framework of the molecular sieve, and the so-called “single” introduction with the formation of Brønsted acid sites occurs. By the SM2 + SM3 mechanism, silicon atoms are introduced in the form of silicate islands of different sizes. It is assumed that weaker acid sites are formed by the SM2 mechanism than by the SM2 + SM3 mechanism [8]. Thus, by regulating the process of introduction of silicon atoms, the acidic properties of silicoaluminophosphate zeolites can be tuned. It is important to note that acid sites in SAPO-n are weaker than in aluminosilicates; such sites are usually called “moderate”.

Among a large number of SAPO-n structures, SAPO-11 molecular sieves (the AEL structural type) stand out due to their relevance in the development of heterogeneous catalysts. These sieves are characterized by a 1-dimensional channel pore system with pore sizes of 4.0 × 6.5 Å and acidic sites of “moderate” strength. This feature allows us to create on its platform, highly selective bifunctional catalysts for the hydroisomerization of n-paraffins C_7+_ [6,9,10] and biofuel production [11,12,13,14,15]. SAPO-11 has demonstrated significant activity and selectivity in the isomerization of n-butene to isobutylene [6] and the isomerization of cyclohexanonoxime to caprolactam [16,17].

The catalytic efficiency of SAPO-11 in the stated processes is influenced by the strength and concentration of acid sites, crystal size, and characteristics of the porous structure. At present, some success has been achieved in the development of ways to regulate the concentration and localization of acid sites [18,19,20,21,22,23], as well as the crystal size and characteristics of the secondary porous structure [24,25,26,27,28,29]. At the same time, despite the advancements in synthesizing SAPO-11 silicoaluminophosphate, the crystallization mechanism remains insufficiently studied, knowledge of which will allow us to move to the directed control of the above-mentioned characteristics of these molecular sieves and the creation of more efficient catalysts based on them [3].

One of the essential differences in the crystallization of AlPO_4_-n and SAPO-n molecular sieves from aluminosilicate zeolites is the formation of intermediate phases in some cases [16,30,31,32,33]. This feature can be a new tool for regulating the properties of SAPO-11 molecular sieves.

In [16], using SAPO-11 zeolite as an example, it was shown that changing the aging temperature of the initial reaction gels can result in the formation of various intermediate phases. Aging of the gel at 90 °C, depending on the aluminum source used (bemite, isopropoxide), led to the formation of hydroaluminophosphate AlPO_4_•2H_2_O or the amorphous phase. At 130 °C, the formation of layered intermediate phases was observed. It is shown that using different intermediate phases, the size and morphology of SAPO-11 crystals can be controlled, resulting in the production of a nanoscale hierarchical porous structure.

In [30], an unidentified crystalline phase was observed in the preparation of reaction gels at room temperature, which transformed into an amorphous phase when heated to 150 °C. During the next two hours, the molecular sieve AlPO_4_-5 was formed. When the crystallization duration was increased to more than 24 h, it transformed into the molecular sieve AlPO_4_-18.

It was shown in [33] that by changing the template content, the nature of the solvent, or adding a silicon source to the reaction mixture, it is possible to synthesize AlPO_4_-5, AlPO_4_-22, AlPO_4_-16, or SAPO-35. It is significant to mention that the studies referenced above practically lack information regarding the impact of the template/Al_2_O_3_ ratio in silicoaluminophosphate reaction gels on the characteristics of the intermediate phases formed during their crystallization, as well as SAPO-11 molecular sieves.

## 2. Materials and Methods

### 2.1. Preparation of Silicoaluminophosphate Gels

For the synthesis of SAPO-11 molecular sieves, we used reaction gels of the following compositions: 1.0Al_2_O_3_•1.0P_2_O_5_•0.2SiO_2_•nDPA•40H_2_O, where *n* is the ratio template/Al_2_O_3_, which was 1.0, 1.4, and 1.8. It is shown by preliminary experiments that higher template contents result in the formation of the SAPO-41 molecular sieve. Al isopropoxide (IPA, 98%, Acros Organics, Noisy-le-Grand, France) and phosphoric acid (H_3_PO_4_, 85%, Reachim, Moscow, Russia) were used as aluminum and phosphorus sources. The silicon source was SiO_2_ sol, obtained according to the method described in [34]. Di-n-propylamine (DPA, 99%, Acros Organics, Schwerte, Germany) was used as the template. The initial reaction gel was prepared as follows: 56 g of distilled water was added to 20.0 g of orthophosphoric acid, which was then added under vigorous stirring depending on the ratio of DPA/Al_2_O_3_ 8.8, 12.2, or 15.8 g DPA. Next, 34.6 g of Al isopropoxide was added to the resulting solution under vigorous stirring until a homogeneous white viscous gel was obtained, into which a calculated amount of SiO_2_ sol was introduced. The reaction gels obtained by the above method at different DPA/Al_2_O_3_ ratios (1.0, 1.4, and 1.8) are designated as Gel-IPA-1.0, Gel-IPA-1.4, and Gel-IPA-1.8, respectively.

### 2.2. Crystallization of Silicoaluminophosphate Molecular Sieves SAPO-11

The crystallization of silicoaluminophosphate gels was carried out at 190 °C without stirring in autoclaves equipped with Teflon cups, and the duration of crystallization varied from 1 to 48 h. The reaction products were separated by centrifugation, washed with distilled water to achieve a neutral pH, and then dried at 100 °C for 48 h. The molecular sieve samples obtained after 48 h of crystallization at the DPA/Al_2_O_3_ ratios of 1.0, 1.4, and 1.8 are hereafter designated as SAPO-11-1.0, SAPO-11-1.4, and SAPO-11-1.8, respectively. Ten autoclaves were used for sequential sampling to study the formation of intermediate phases and crystallization kinetics. During the first 10 h, one autoclave was removed from the oven every two hours, then after 4 h until reaching 24 h, and after 24 h until reaching 48 h.

### 2.3. Characterization

The phase composition of dried gels, intermediate phases, and molecular sieves was studied on a Shimadzu XRD 7000 diffractometer (Shimadzu Corporation, Kyoto, Japan) in CuKα radiation. Scanning was carried out in the region of 2θ angles from 3 to 40° with a step of 1° min. The processing of X-ray images and phase analysis were performed using the Shimadzu XRD program (version 7.04) and the PDF2 database (version 2.2201). Crystallinity was estimated by the content of an amorphous halo in the region from 20 to 30° 2θ in the Shimadzu XRD Crystallinity program (version 7.04).

^31^P and ^27^Al MAS NMR spectra of dried gels and intermediate phases were recorded on a BRUKER AVANCE-II 400 WB (Bruker Corporation, Billerica, MA, USA) spectrometer using a 4 mm H/X MAS WVT probe with operating frequencies of 162.0 and 104.2 MHz, respectively, and a MAS frequency of 12 kHz. To take VSMR NMR spectra on ^31^P nuclei, we used a single-pulse technique (90-degree pulse) with the following imaging parameters: pulse duration—2.1 μs; number of repetitions—32; time between repetitions—120 s.

Raman spectra of the intermediate phases were recorded on a FT-Raman NXR 9650 FT-Raman spectrometer (Thermo Fisher Scientific, Waltham, MA, USA) in the 70–800 cm^−1^ region with a resolution of 1 cm^−1^.

The microstructure of intermediate phases was studied by transmission electron microscopy (TEM) on a Hitachi HT7700 electron microscope (Hitachi High-Tech Corporation, Tokyo, Japan) in the light field mode at an accelerating voltage of 100 kV.

SAPO-11’s crystal sizes and morphology were studied by scanning electron microscopy (SEM) on a Hitachi Regulus SU8220 electron microscope (Hitachi High-Tech Corporation, Tokyo, Japan) in the secondary electron registration mode at an accelerating voltage of 5 kV.

The characteristics of the porous structure (the specific BET surface area and volume of micro- and mesopores) were measured by using the low-temperature adsorption–desorption method N_2_ on a Quantachrome Nova 1200e sorbtometer (Quantachrome Instruments, Boynton Beach, FL, USA). The volume of micropores in the presence of mesopores was estimated by the *t*-plot method. The pore size distribution was calculated using the BJH model (Barrett–Joyner–Halendy) along the desorption branch.

The elemental composition of the calcined silicoaluminophosphate molecular sieves was carried out by X-ray fluorescence spectroscopy on a Shimadzu EDX-7000P (Shimadzu Corporation, Duisburg, Germany) spectrometer.

The type and concentration of acidic sites were evaluated by IR spectroscopy with pyridine adsorption. The IR spectra of adsorbed pyridine were recorded on a Bruker Vertex-70V IR spectrometer (Bruker Optic GmbH, Ettlingen, Germany) with a resolution of 4 cm^−1^. The samples were calcined at 450 °C under vacuum for 2 h. Pyridine was adsorbed at 150 °C for 30 min, then evacuated at 150 °C for 30 min. IR spectra were obtained at room temperature before the adsorption and after desorption of pyridine at 150 °C, 250 °C, and 350 °C at intervals of 30 min. The concentration of Brønsted acid sites (BASs) and Lewis acid sites (LASs) was estimated according to the method described in [35] by integrating the absorbance bands at 1545 and 1454 cm^−1^, respectively.

### 2.4. Hydroisomerization of n-Hexadecane

N-hexadecane (n-C_16_H_34_, 99%, Acros Organics, Morris Plains, NJ, USA) was used as a model feedstock. Its hydroisomerization was studied in a flow reactor at 280–340 °C, with a pressure of 3.0 MPa, a molar ratio of H_2_/n-C_16_H_34_ = 10, and a mass feed rate of feedstock (WHSV)of 2 h^−1^. The reaction products were analyzed by gas–liquid chromatography on the Chromatec-Crystal 5000 chromatograph (Chromatec, Yoshkar-Ola, Russia) with a flame ionization detector (50 m glass capillary column, SE-30). The identification of the products was carried out through gas chromatography–mass spectrometry on a Shimadzu GCMS-TQ8050NX (Shimadzu Corporation, Kyoto, Japan).

## 3. Results and Discussion

### 3.1. The Effect of the DPA/Al_2_O_3_ Ratio on the Formation of Intermediate Phases and Crystallization of SAPO-11

As noted above, in the works devoted to the study of crystallization of molecular sieves with an AEL structure, no proper attention was paid to the study of the properties of the formed reaction gels and their influence on the properties of crystallization products.

**Figure 1:**
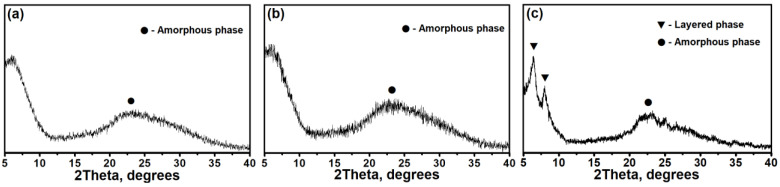
X-ray diffraction patterns of silicoaluminophosphate gels prepared at different DPA/Al_2_O_3_ ratios: (**a**) Gel-IPA-1.0 sample, (**b**) Gel-IPA-1.4 sample, and (**c**) Gel-IPA-1.8 sample.

Figure 1 shows the X-ray diffraction patterns of Gel-IPA-1.0, Gel-IPA-1.4, and Gel-IPA-1.8. It is evident from the patterns that Gel-IPA-1.0 and Gel-IPA-1.4 exhibit a characteristic halo in the range of 15 to 30° 2ϴ, indicating an amorphous nature. For the Gel-IPA-1.8 sample, signals at 6.5 and 8.0° 2ϴ are observed, indicating the formation of a mixture of amorphous and layered phases. It was previously shown [36] that layered materials are ordered only along the a–b plane direction and are thin 2D plates bound by weak Van der Waals forces. The XRD data reveal that the interplanar distances (d) observed in the layered phases, characteristic of the peaks at 6.2° (d = 14.0 Å) and 8.0° (d = 11.0 Å), are close to 6.5° (d~13.6 Å) and 8.1° (d~10.9 Å) in the (100) and (110) planes of the AEL structure. These findings suggest the presence of common structural fragments in layered silicoaluminophosphate and SAPO-11 molecular sieves.

**Figure 2:**
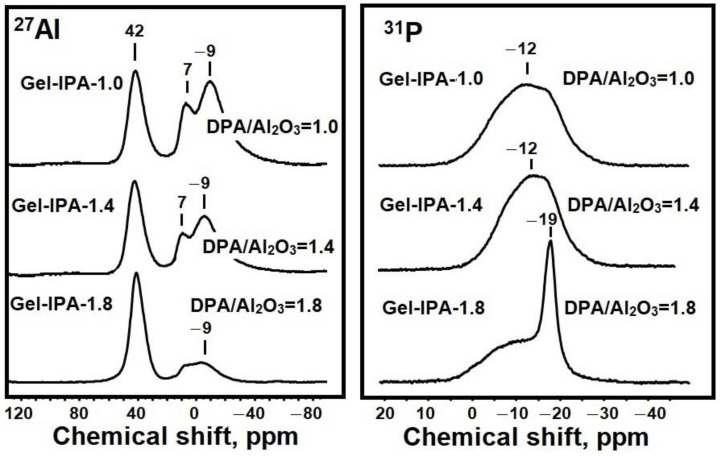
MAS NMR spectra of ^27^Al and ^31^P silicoaluminophosphate gels prepared at different DPA/Al_2_O_3_ ratios.

To better understand the properties of the formed reaction gels, ^27^Al and ^31^P MAS NMR spectra were recorded and can be seen in Figure 2. The 27Al MAS NMR spectra show three main signals at 42, 7, and −9 ppm. The signal at 42 ppm is typically attributed to Al atoms with oxygen surroundings [AlO_4_] in amorphous silicoaluminophosphate, and the signal at 7 ppm is attributed to Al atoms with oxygen surroundings [AlO_4_] in amorphous silicoaluminophosphate. The signal at −9 ppm was attributed to aluminum atoms [AlO_6_] in amorphous silicoaluminophosphate [36,37,38]. With an increasing template content (DPA/Al_2_O_3_ ratio), the signal at 42 ppm is enhanced, and the signals at 7 and −9 ppm are weakened. The results indicate a deeper interaction between phosphorus and aluminum compounds with the formation of ≡Al-O-P≡ bonds and the formation of silicoaluminophosphate. In the ^31^P spectra of the Gel-IPA-1.0 and Gel-IPA-1.4 samples, a broad signal with a maximum at −12 ppm is observed, which can be attributed to amorphous silicoaluminophosphate of the type P(OAl)n(H_2_O)_4−n_ with n = 1–4. An additional signal at −19 ppm appears in the spectrum of the Gel-IPA-1.8 sample, which is attributed to phosphorus atoms [PO_4_] in the layered phase [36,37,38].

Analyses of the ^27^Al and ^31^P MAS NMR spectra of prepared gels at different DPA/Al_2_O_3_ ratios show that the interaction between phosphorus and aluminum compounds with the formation of silicoaluminophosphate increases with an increasing DPA/Al_2_O_3_ ratio. In addition, when aluminum isopropoxide is used as a source of aluminum, a preferential formation of aluminophosphate P(OAl)n(H_2_O)_4−n_with n = 1–4 is already observed at the initial stage of gel preparation compared to the less reactive aluminum sources such as boehmit [39].

**Figure 3:**
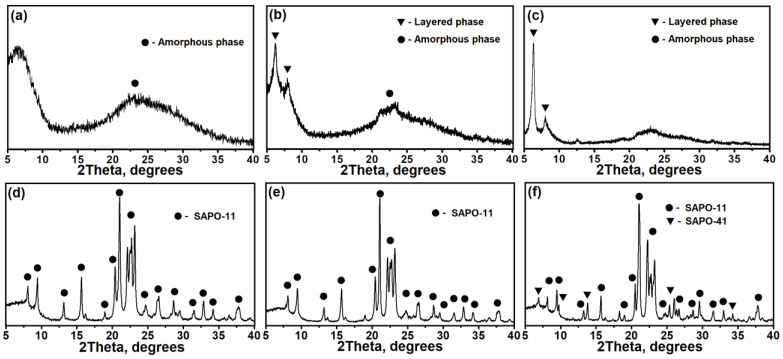
X-ray diffraction patterns of intermediate phases and molecular sieves prepared at different DPA/Al_2_O_3_ ratios: (**a**) SAPO-IF-1.0, (**b**) SAPO-IF-1.4, (**c**) SAPO-IF-1.8, (**d**) SAPO-11-1.0, (**e**) SAPO-11-1.4, and (**f**) SAPO-11-1.8.

Figure 3 shows the results of the crystallization of reaction gels prepared at different DPA/Al_2_O_3_ ratios. After 1 h from the beginning of crystallization, the formation of intermediate phases is observed, the composition of which depends on the DPA/Al_2_O_3_ ratio. At the crystallization of the Gel-IPA-1.0 gel, the phase composition does not change; the intermediate amorphous phase (IF-IPA-1.0) remains. At the crystallization of Gel-IPA-1.4 gel, the formation of a mixture of amorphous and layered phases is observed (IF-IPA-1.4), and at the crystallization of Gel-IPA-1.8 gel, mainly layered silicoaluminophosphate is formed (IF-IPA-1.8). After 12 h of crystallization, a complete transformation of intermediate phases into SAPO-11 molecular sieves is observed; after 24 h, the latter are characterized by maximum crystallinity, more than 90%. At DPA/Al_2_O_3_ ratios of up to 1.4 in the reaction gels, the main product of crystallization is molecular sieve SAPO-11; at a DPA/Al_2_O_3_ ratio equal to 1.8, small impurities of molecular sieve SAPO-41 are formed. The main reason for the formation of SAPO-41 molecular sieve impurities is the increased content of the template (DPA/Al_2_O_3_ ratio) in the reaction mass, which promotes the formation of molecular sieves with an AFO structure.

**Figure 4:**
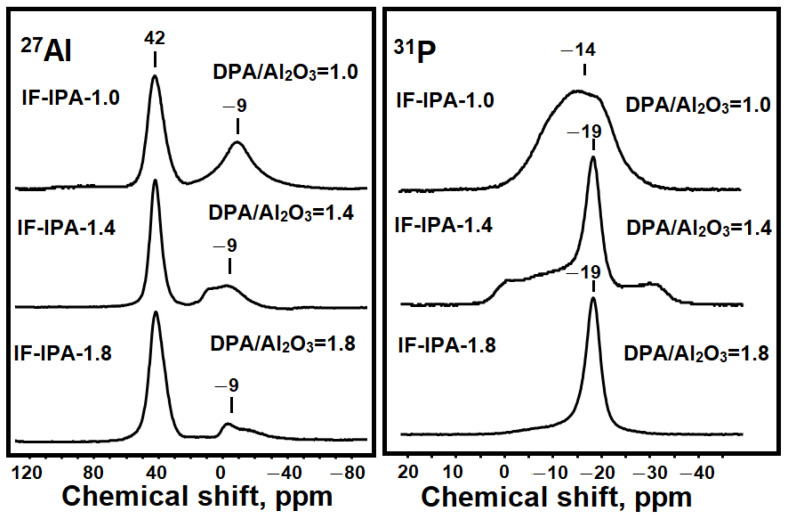
^27^Al and ^31^P MAS NMR spectra of intermediate silicoaluminophosphate phases.

The microstructure of the formed intermediate phases was further studied by ^27^Al and ^31^P MAS NMR, Raman spectroscopy, and TEM. The ^27^Al and ^31^P MAS NMR spectra of the forming intermediate phases are shown in Figure 4. In the spectra of the ^27^Al intermediate phase IF-IPA-1.0, signals at 42 and −9 ppm are observed, which are attributed to Al atoms with [AlO_4_] and [AlO_6_] surroundings in amorphous silicoaluminophosphate [36,37,38,39]. The spectrum of ^31^P IF-IPA-1.0 shows a shift of the maximum compared to the spectrum of the Gel-IPA-1.0 reaction gel from −12 to −14 ppm, which indicates the formation of aluminophosphate P(OAl)n(H_2_O)_4−n_ with n = 1–4 with a higher proportion of ≡Al-O-P≡ type bonds. In the spectrum of the ^27^Al intermediate phase IF-IPA-1.4, a signal enhancement at 42 ppm is observed. In the ^31^P spectrum, the appearance of a new signal at −19 ppm is characteristic, as noted above, for layered silicoaluminophosphate. In the spectrum of the ^27^Al layered phase, a major signal at 42 ppm is observed, while the ^31^P spectrum exhibits signals at −19 and −30 ppm, which are attributed to [AlO_4_] and [PO_4_] in crystalline aluminophosphate [36,37,38,39].

**Figure 5:**
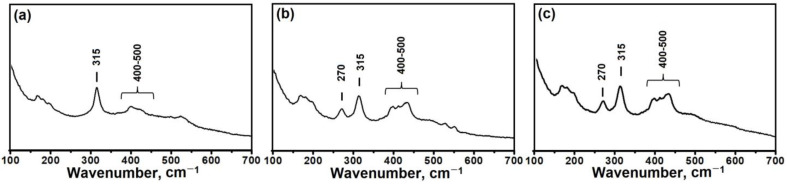
Raman spectra of intermediate phases: (**a**) IF-IPA-1.0, (**b**) IF-IPA-1.4 и, and (**c**) IF-IPA-1.8.

As noted above, it is possible for intermediate phases to contain fragments in common with molecular sieves, such as secondary building units (SBUs). The Raman spectra of the isolated intermediate phases are shown in Figure 5. It can be seen that the spectra of the layered phases IF-IPA-1.4 and IF-IPA-1.8 contain major bands at 270, 315, and 400–500 cm^−1^. The 270 cm^−1^ band is typically associated with vibrations in 10-R rings, the bands at 400–500 cm^−1^ correspond to 4-R and 6-R rings, and the 315 cm^−1^ band to [AlO_4_] tetrahedra in molecular sieves [40,41]. In the spectrum of the intermediate phase IF-IPA-1.0, the band at 270 cm^−1^ is absent because it contains no 10-R rings and fewer building fragments in common with molecular sieves.

A comparison of the Raman spectroscopy and the ^27^Al and ^31^P MAS NMR results show that the layered phases have a close structure to the silicoaluminophosphate molecular sieves of SAPO-11, as they share common structural fragments: 4-R, 6-R, and 10-R rings. The amorphous phase also contains common structural fragments with SAPO-11; however, their share is significantly lower than for the layered phases.

**Figure 6:**
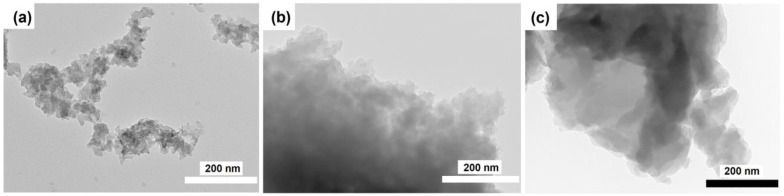
TEM images of intermediate phases: (**a**) IF-IPA-1.0, (**b**) IF-IPA-1.4 и, and (**c**) IF-IPA-1.8.

Figure 6 shows TEM images of the intermediate phases. It can be seen that the intermediate phase IF-IPA-1.0 resembles, in structure, a xerogel formed from aggregates of spherical amorphous particles ranging in sizes from 5 to 10 nm. The sample of the intermediate phase IF-IPA-1.4 is a mixture of ~300 nm plates and spherical particles of 5 to 10 nm in size. Comparing the results obtained with XRD data, it can be concluded that the spherical particles are amorphous silicoaluminophosphate, and the plates are in a layered phase. The intermediate layered phase IF-IPA-1.8 consists of thin plates with a size of ~100–300 nm.

Thus, by varying the template content in the reaction gels prepared using aluminumisopropoxide, it is possible during crystallization to form intermediate phases that differ significantly in microstructure, phase composition, morphology, and the size of the particles of which they are composed. The main reason for the formation of different intermediate phases is that the amount of template (amine) has a significant impact on the pH value of the reaction medium. For the initial reaction gels, increasing the DPA/Al_2_O_3_ ratio from 1.0 to 1.8 results in an increase of the pH from 7.1 to 8.2. When the pH is lower, amorphous intermediate phases tend to form, while an elevation of the pH leads to the development of layered zeolite-like intermolecular phases.

**Figure 7:**
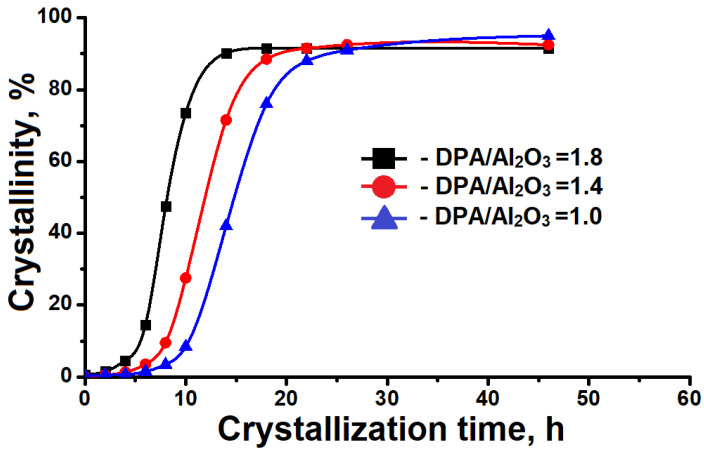
Crystallization kinetics of reaction gels with different ratios of DPA/Al_2_O_3_ into the SAPO-11 molecular sieve.

The template content in the reaction gels also has a significant role in the crystallization kinetics of SAPO-11 molecular sieves. Figure 7 displays the crystallization kinetic curves of reaction gels with different DPA/Al_2_O_3_ ratios. It is noticeable that the crystallization of SAPO-11 molecular sieves proceeds faster when intermediate layered phases are formed. The results obtained are attributed to the fact that the layered phase contains more SBUs (decipher), such as 4-R, 6-R, and 10-R rings, from which the molecular sieves are formed. This feature facilitates the formation of nuclei of future SAPO-11 crystals, thereby accelerating the crystallization. The results obtained indicate that the intermediate phases contain structurally similar secondary building units that are similar to SAPO-11 molecular sieves. It is possible that their crystallization occurs through the crystallization-by-particle attachment (CPA) mechanism rather than the monomer-by-monomer mechanism [42].

### 3.2. The Effect of Intermediate Phases on the Properties of SAPO-11 Molecular Sieves

**Figure 8:**
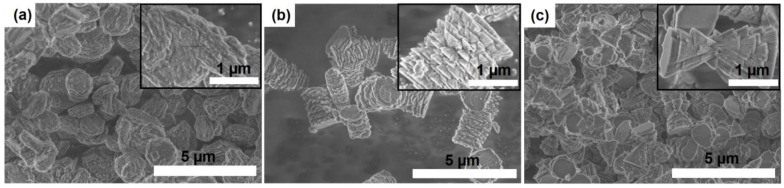
SEM images of silicoaluminophosphate molecular sieves: (**a**) Sample SAPO-11-1.0, (**b**) sample SAPO-11-1.4, and (**c**) sample SAPO-11-1.8.

The morphology and crystal size of SAPO-11 molecular sieves are among the key characteristics affecting their adsorption and catalytic properties. Figure 8 shows SEM images of silicoaluminophosphate molecular sieves synthesized from gels with different ratios of DPA/Al_2_O_3_. The SAPO-11 sample synthesized from the Gel-IPA-1.0 gel has spherical-like aggregates (1–1.5 μm) formed from crystals as thin plates of ~100 nm. In the Gel-IPA-1.4 gel, silicoaluminophosphate sieve SAPO-11 aggregates are formed in the form of cylinders of ~1–2 μm, which are formed from cones of ~200–300 nm, and in the Gel-IPA-1.8 gel, aggregates with the morphology of conjoined pyramids are ~1–1.5.

In molecular sieves with a one-dimensional porous structure, the channels are usually directed along the longest face [9]. It follows from our results that the aspect ratio of primary crystals (the ratio of crystal length to diameter) increases as the DPA/Al_2_O_3_ ratio in the initial gels increases from 1.0 to 1.8. Thus, by changing the DPA/Al_2_O_3_ ratio from 1.0 to 1.8 in the reaction gels prepared using aluminum isopropoxide, it is possible to control the morphology and dispersity of crystals during the formation of the SAPO-11 molecular sieve.

**Figure 9:**
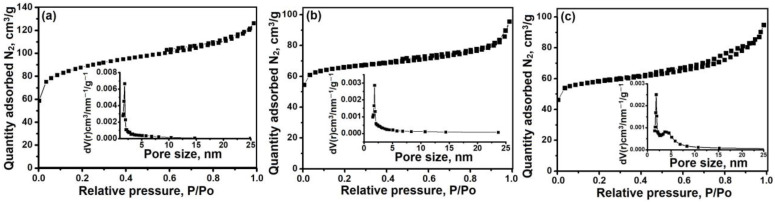
Nitrogen adsorption–desorption isotherms and pore size distribution of silicoaluminophosphate molecular sieves synthesized from gels with different ratios DPA/Al_2_O_3_: (**a**) Sample SAPO-11-1.0, (**b**) sample SAPO-11-1.4, and (**c**) sample SAPO-11-1.8.

Nitrogen adsorption–desorption isotherms and the pore size distribution for the SAPO-11 molecular sieve samples prepared from reaction gels with different template contents are shown in Figure 9, and Table 1 presents the porous structure characteristics. It can be seen that all SAPO-11 samples exhibit type IV isotherms with the H3-type hysteresis loop that characterizes mesoporous materials. Mesopores with sizes from 2 to 25 nm are formed in this sample by the incomplete fusion of nanoscale crystals. With an increasing DPA/Al_2_O_3_ ratio, a decrease in the specific surface area and volume of mesopores is observed due to an increase in the size of primary nanoscale crystals from which spherical aggregates are formed. Consequently, when using its isopropoxide as a source of aluminum, changing the ratio of DPA/Al_2_O_3_ in the reaction mixture, it is possible to regulate the porous structure of the SAPO-11 molecular sieve and synthesize samples of SAPO-11 molecular sieves with a hierarchical porous structure.

**Figure 10:**
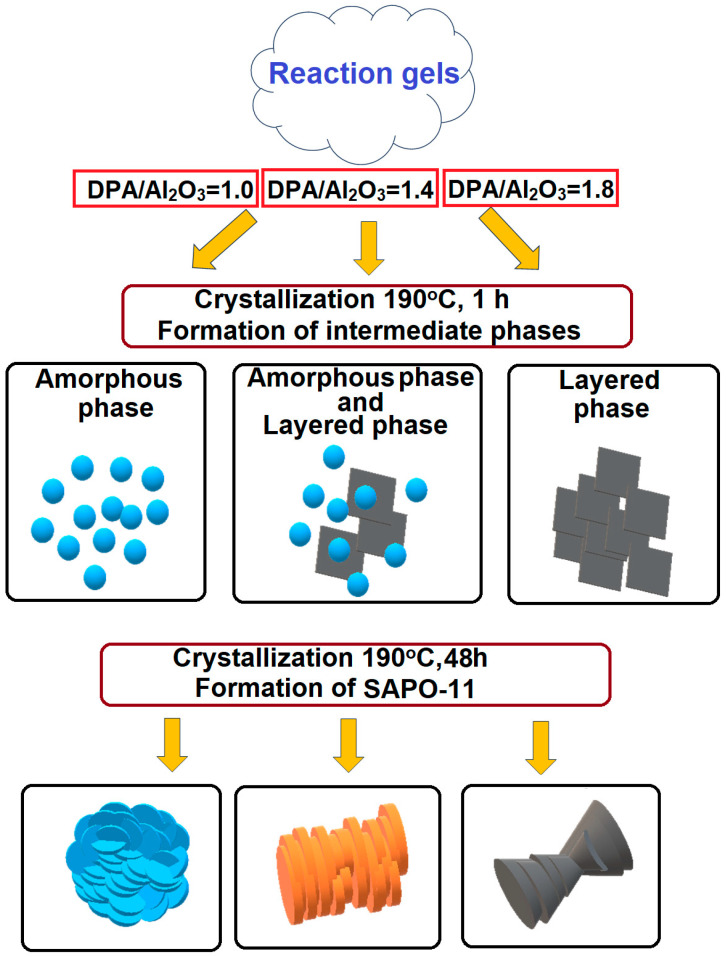
Schematic of crystallization of SAPO-11 silicoaluminophosphate molecular sieve from gels with different ratios of DPA/Al_2_O_3_.

A generalized scheme of the influence of the DPA/Al_2_O_3_ ratio in the reaction gels on the crystallization route and characteristics of the resulting SAPO-11 molecular sieves is shown in Figure 10.

The acidity of SAPO-n molecular sieves significantly depends on the amount of silicon in their crystal lattice [3,6]. Table 2 shows the results of the elemental analysis of reaction gels and SAPO-11 samples synthesized at different ratios of DPA/Al_2_O_3_. It can be seen that the reaction gels have a higher silicon content than the SAPO-11 molecular sieves. Differences in the silicon content between the reaction gels and crystalline silicoaluminophosphate are due to the fact that part of the silicon during crystallization is not incorporated into the SAPO-11 lattice and remains in the mother liquor. There is a slight increase in the silicon content with an increasing ratio of DPA/Al_2_O_3_, which is due to the improvement in its incorporation into the lattice with an increasing template content [43].

**Figure 11:**
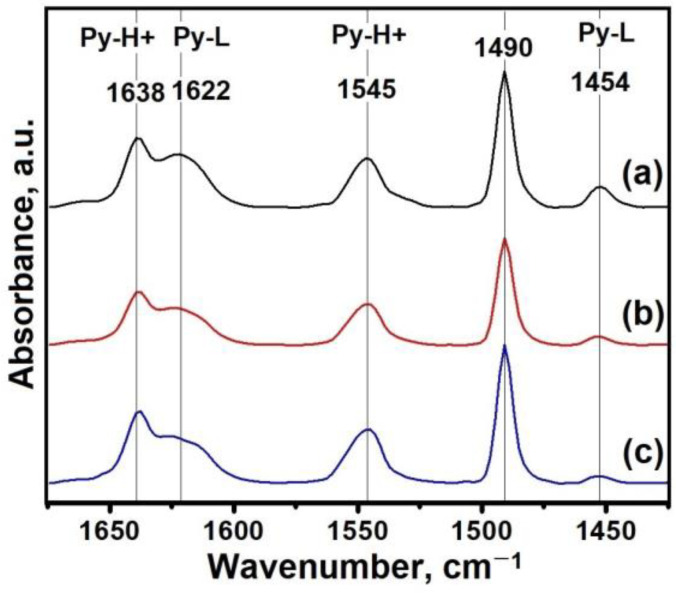
IR spectra of adsorbed pyridine on SAPO-11 samples: (**a**) Sample SAPO-11-1.0, (**b**) sample SAPO-11-1.4, and (**c**) sample SAPO-11-1.8.

As already mentioned, silicoaluminophosphate molecular sieves SAPO-n are characterized by their weaker strength compared to aluminosilicate zeolite acid sites, which are called “moderate”. The IR spectra of adsorbed pyridine over the SAPO-11 samples synthesized with different ratios of DPA/Al_2_O_3_ are shown in Figure 11, and Table 3 shows the concentration values of the acid sites. All samples exhibit three absorbance bands at 1455, 1490, and 1545 cm^−1^. The absorbance bands at 1545 cm^−1^ and 1455 cm^−1^ are assumed to be attributed to the pyridine molecules adsorbed on the BAS and LAS acid sites, respectively. The band at 1490 cm^−1^ is attributed to the pyridine molecules adsorbed on both types of acid sites [35]. As the DPA/Al_2_O_3_ ratio increases, a change in the concentration of both types of acid sites is observed in the reaction gels. These results can be explained by the fact that the DPA/Al_2_O_3_ ratio has a complex effect on the silicon content and the size of the primary crystals, on which the concentrations of acid centers can strongly depend. It can be seen that for the SAPO-11-1.8 sample, the highest total concentration of both types of acid centers is observed, which is due to the highest silicon content. The SAPO-11-1.0 sample is characterized by a higher concentration of acid centers despite the close silicon content with SAPO-11-1.4, which is due to the smallest size of its crystals, which provides better access of pyridine molecules to the acid centers.

### 3.3. The Catalytic Properties of SAPO-11 in the Hydroisomerization of n-C_16_

It was previously shown [44] that in bifunctional catalysts for the hydroisomerization of n-paraffins using SAPO-11 molecular sieves as the basis, it is necessary to provide a Pt content of at least 0.5 wt% in order to limit the limiting stages of catalytic transformations at the acidic sites. It was found that during the hydroisomerization of n-hexadecane on SAPO-11 samples containing the specified amount of Pt, the primary products are C_16_ isomers with one and two methyl groups. Among monomethyl substituted isomers, the main products are 2-, 3-, 4-, 5, 6-, 7-, and 8-monomethyl pentadecanes (Table 4), with the contents of 2- and 3-monomethyl pentadecane up to 16.5%. The predominant formation of monomethyl pentadecane with the terminal methyl groups is explained by the fact that n-hexadecane is hydroisomerized by the PMKLS “pore mouth and key-lock selectivity” mechanism when its molecules are adsorbed in single mouths (PM) of 1D-10R channels [45]. The formation of (2–5)-monomethylpentadecanes is explained by the fact that part of the n-paraffin molecules is converted by the FEL “free energy landscape approach” mechanism inside the pores. Among the dimethyl-substituted isomers of C_16_, the main reaction products are 2,12- or 2,6-dimethyltetradecanes (Table 4), and, in addition, the formation of monoethyl isomers of C_16_.

**Figure 12:**
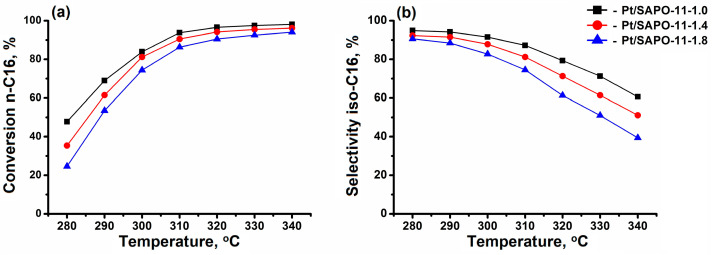
Hydroisomerization of n-hexadecane on 0.5% wt.% Pt/SAPO-11 samples: (**a**) Conversion of n-hexadecane, and (**b**) selectivity of formation of C_16_ isomers.

Increasing the temperature of the catalytic transformations of n-hexadecane from 280 to 340 °C results in enhanced conversion and a reduced total selectivity for isomers C_16_ due to a greater contribution of the side reaction of hydrocracking (Figure 12). The main products of hydrocracking at low degrees of conversion are the alkanes C_7_–C_13_ and, at higher degrees, C_3_–C_4_.

The highest activity and selectivity are shown by the sample Pt/SAPO-11-1.0, which is characterized by nanosized crystals with a low aspect ratio and a higher concentration of available acid sites (Figure 12). Due to this porous structure, diffusion limitations for reacting molecules are reduced, leading to a decrease in their residence time inside the channels and the contribution of the secondary cracking reaction on the acid sites [46,47,48]. For the Pt/SAPO-11-1.8 sample, the lowest selectivity for the C_16_ isomers is observed due to the presence of primary crystals with higher aspect ratios, in which the residence time of molecules in the channels is significantly increased, and the contribution of cracking reactions is increased.

Thus, the DPA/Al_2_O_3_ ratio significantly affects the characteristics of the SAPO-11 molecular sieve crystals, on which the activity and selectivity in the hydroisomerization of n-hexadecane of bifunctional catalysts prepared on their basis strongly depend. Therefore, a hierarchical porous structure formed from nanoscale crystals with a low aspect ratio is required in high-performance catalysts. Obtaining such SAPO-11 samples is achieved by the crystallization of the amorphous silicoaluminophosphate intermediate phase. The results obtained suggest that when creating industrial catalysts for the isodeparaffinization of fuels and oils using SAPO-11, it is important to consider the ratio of SDA to Al_2_O_3_ in the reaction gel, which allows for a fine-tuning of their porous structure and acidity. These results may also be useful for developing other catalytic systems using SAPO-11, such as the isomerization of olefins and cyclohexanone oxime, as well as the methylation of aromatic hydrocarbons.

## 4. Conclusions

The influence of the di-n-propylamine/Al_2_O_3_ ratio in the reaction silicoaluminophosphate gels, which were prepared using Al isopropoxide as an aluminum source, on the characteristics of the intermediate phases and SAPO-11 molecular sieves formed during their crystallization was found using XRD, XRF, MAS NMR ^27^Al, and ^31^P Raman spectroscopy, as well as the SEM, TEM, and adsorption–desorption N_2_ techniques. After one hour of crystallization at 190 °C using reaction gels with DPA/Al_2_O_3_ ratios equal to 1.0, 1.4, and 1.8, the following were formed: Amorphous silicoaluminophosphate consisting of aggregates of spherical particles with sizes from 5 to 10 nm, a mixture of amorphous and layered phases, and a layered phase representing thin plates of ~300 nm, respectively.

- Intermediate phases with a layered structure crystallize into SAPO-11 molecular sieves faster than amorphous systems due to the closeness of their structure to molecular sieves.
- The di-n-propylamine/Al_2_O_3_ ratio in the reaction gels affects the morphology and size of primary SAPO-11 crystals. It was found that using isopropoxide as an aluminum source allows the preparation of SAPO-11 with a hierarchical porous structure. SAPO-11 samples synthesized at a DPA/Al_2_O_3_ ratio = 1.0 are characterized by S_BET_ = 286 m^2^/g and V_meso_ = 0.13 cm^3^/g and are spherical-like aggregates (1–1.5 μm) of primary crystals in the form of thin plates of ~100 nm. The SAPO-11 samples prepared at DPA/Al_2_O_3_ = 1.4 represent conglomerates in the form of cylinders of ~1–2 μm formed from mated cones ~200–300 nm and are characterized by S_BET_ = 209 m^2^/g and V_meso_ = 0.08 cm^3^/g. With a further increase in the DPA/Al_2_O_3_ ratio of up to =1.8, crystals in the form of conjoined pyramids of ~1–1.5 μm in size are formed and characterized by S_BET_ = 184 m^2^/g and V_meso_ = 0.09 cm^3^/g.
- The synthesized DPA/Al_2_O_3_ ratio = 1.0 sample SAPO-11, with nanocrystals of cubic morphology, provides higher values of n-hexadecane conversion and selectivity for C_16_ isomers due to smaller crystal sizes, which reduce the diffusion limitations and decrease the residence time of the reaction products inside the channels compared to the samples with larger crystals.

The obtained results demonstrate the feasibility of controlling the morphology and crystal sizes of SAPO-11 molecular sieves to develop bifunctional catalytic systems. By altering the DPA/Al_2_O_3_ ratio in the reaction gels, it is possible to achieve varied activity and selectivity for the hydroisomerization of higher n-paraffins C_16+_.

## Figures and Tables

**Table 1:** Characteristics of the porous structure of samples of silicoaluminophosphate molecular sieve SAPO-11.

Sample	S_BET_, m^2^/g	S_EX_, m^2^/g	V_micro_, cm^3^/g	V_meso_, cm^3^/g
SAPO-11-1.0	286	172	0.06	0.13
SAPO-11-1.4	209	66	0.07	0.08
SAPO-11-1.8	184	65	0.06	0.09

Symbols: S_BET_—specific surface according to BET; S_EX_—external specific surface area; V_micro_—specific volume of micropores; V_Ʃ_—total pore volume.

**Table 2:** Chemical compositions of reaction gels and their crystallization products.

Sample	Chemical Composition, Gel	Chemical Composition, AEL
SAPO-11-1.0	Al_1.00_P_0.98_Si_0.09_	Al_1.00_P_0.98_Si_0.06_
SAPO-11-1.4	Al_1.00_P_0.98_Si_0.10_	Al_1.00_P_0.98_Si_0.07_
SAPO-11-1.8	Al_1.00_P_0.99_Si_0.09_	Al_1.00_P_0.99_Si_0.08_

**Table 3:** Concentrations of acid sites according to IR spectroscopy data with pyridine adsorption.

Sample	Acidity (μmol/g)					
	BAS			LAS		
	150 °C	250 °C	350 °C	150 °C	250 °C	350 °C
SAPO-11-1.0	155	90	30	43	13	6
SAPO-11-1.4	130	89	27	19	7	6
SAPO-11-1.8	173	127	37	13	6	5

**Table 4:** Hydroisomerization results of n-hexadecane at 300 °C over Pt-containing SAPO-11 samples.

Catalyst	Pt/SAPO-11-1.0	Pt/SAPO-11-1.4	Pt/SAPO-11-1.8
Conversion, wt %	83.9	81.2	74.4
Selectivity by iso-C_16_, wt %	94.2	87.8	82.7
Iso-C_16_ yield, wt %:			
2-MeC_15_	8.0	5.5	6.2
3-MeC_15_	8.5	5.9	7.4
4-MeC_15_	6.8	4.8	5.8
5-MeC_15_	6.2	4.8	4.8
6+-MeC_15_	22.4	15.6	21.5
(CH_3_)_2_–C_14_	23.0	34.5	15.7
Yield ΣC_1_–C_4_, wt %	0.5	0.5	0.6
Yield ΣC_5_–C_15_, wt %	7.1	9.4	12.3

## Data Availability

Data are contained within the article.
